# School Psychological Environment and Learning Burnout in Medical Students: Mediating Roles of School Identity and Collective Self-Esteem

**DOI:** 10.3389/fpsyg.2022.851912

**Published:** 2022-06-02

**Authors:** Wanwan Yu, Shuo Yang, Ming Chen, Ying Zhu, Qiujian Meng, Wenjun Yao, Junjie Bu

**Affiliations:** ^1^The Second Clinical Medical School, Anhui Medical University, Hefei, China; ^2^Department of Radiology, Anhui Mental Health Center, Hefei, China; ^3^School of Biomedical Engineering, Anhui Medical University, Hefei, China; ^4^Department of Radiology, The Second Affiliated Hospital of Anhui Medical University, Hefei, China

**Keywords:** school psychological environment, school identity, collective self-esteem, learning burnout, medical students

## Abstract

Learning burnout is an important indicator that reflects an individual’s learning state. Understanding the influencing factors and mechanism of learning burnout of medical students has practical significance for improving their mental health. This study aimed to explore the mediating roles of school identity and collective self-esteem between school psychological environment and learning burnout in medical students. A total of 2,031 medical students (942 men and 1,089 women, age range: 17–23 years) were surveyed using the School Psychological Environment Questionnaire (SPEQ), School Identity Questionnaire (SIQ), Collective Self-esteem Scale (CSES), and Learning Burnout Scale (LBS). The results showed the following: (1) school psychological environment had a negative effect on learning burnout among medical students (β = −0.19, *p* < 0.001), and (2) school identity and collective self-esteem played significant mediating roles between school psychological environment and learning burnout [95% CI = (−0.43, −0.31)]. Specifically, there were three paths that school psychological environment and learning burnout: first, through the independent mediating role of school identity; second, through the independent mediating role of collective self-esteem; and third, through the chain mediating roles of school identity and collective self-esteem. The findings reveal that school psychological environment not only directly influences the learning burnout of medical students but also indirectly influences it through school identity and collective self-esteem. Thus, this study has some important implications for prevention and intervention of learning burnout among medical students.

## Introduction

Learning burnout refers to negative attitudes and behaviors resulting from excessive pressure or lack of interest in learning, often accompanied by anxiety, fatigue, depression, and low self-esteem (Lian et al., 2005). In addition to affecting academic performance (May et al., 2015), learning burnout can also lead to psychological and behavioral problems, such as dropping out, eating disorders and a decline in mental health (Bask and Salmela-Aro, 2013; Saule et al., 2014; Kristanto et al., 2016; Liu et al., 2020). College students have a high incidence of learning burnout. A survey of college students found that the incidence of middle-to-high learning burnout was approximately 46% (Meriläinen, 2014). In China, the detection rate of college students’ learning burnout can reach up to 50% or more (Li and Tan, 2007). Medical students are a relatively special group of college students, with long training periods and heavy learning tasks, which are more prone to learning burnout (Zhai et al., 2014). Academic burnout, as an important indicator reflecting the negative learning psychology of individuals, is also an important factor affecting the quality of learning. The discussion on the influencing factors and mechanism of academic burnout of medical students can provide theoretical basis for relevant educational intervention measures. Although many studies have focused on the influencing factors of learning burnout, there is not enough research on this group of medical students (Lee et al., 2017; Moghadam et al., 2020; Yun et al., 2020; Rana et al., 2021). Therefore, it is imperative to explore the influencing factors and mechanisms of medical students’ learning burnout.

School is an important place for students to live and study; moreover, it acts as an important microsystem that affects individual growth and development (Bronfenbrenner, 1979; Eccles and Roeser, 2011). As a complex multidimensional structure, school psychological environment includes the characteristics of the school’s environmental norms, goals, values, interpersonal relationships, teaching practices, and organizational structure (Cohen et al., 2009). The stage-environment matching theory states that when the school environment meets the development needs of students, it is conducive to promoting their physical and mental health. However, when the school environment does not meet these development needs, it easily causes internal and external problems, including feelings of inferiority, depression, and even engaging in criminal activity (Geldhof et al., 2013). Empirical studies have found that a positive and supportive school psychological environment can help students achieve higher academic performance (Zhou et al., 2016) and reduce the occurrence of problematic behaviors such as bullying, suicidal ideation, and game addiction (Li et al., 2016; Pfledderer et al., 2019; Rezapour et al., 2019). A study of primary and middle school students found that school psychological environment and learning burnout were significantly negatively correlated (Wu et al., 2012; Zhang and Gao, 2019). At present, the research on the relationship between school psychological environment and learning burnout mainly focuses on the group of primary and middle school students, and rarely involves the group of college students. Although higher education and primary education have their own uniqueness, there is an inherent consistency between university campuses and primary and secondary schools, which is mainly reflected in school norms and disciplines, teacher-student relationships, classmate relationships, and material environment (Zhu, 2018). Both are important components of school psychological environment (Fraser et al., 1986; Zhou et al., 2016). Based on these studies, the first proposed hypothesis is: school psychological environment significantly negatively predicts the learning burnout of medical students.

Although the relationship between the school psychological environment and learning burnout has been verified by some researchers, the mechanism by which school psychological environment affects learning burnout still needs to be further explored. School identity indicates that an individual recognizes that they belong to the school group and realizes that becoming a member of this school will influence their own sense of self-worth and how they come to understand their own emotions (Osterman, 2000). According to the situation-process-outcome model, the influence of situational factors on an individual’s development can occur through internal psychological processes (Roeser et al., 1996). School identification can be regarded as a mechanism or process that acts as a basis for the school’s psychological environment; further, it influences an individual’s emotions, attitudes, and behaviors (Reynolds et al., 2015). School identity can help reduce learning burnout. Huang et al. (2016) pointed out that school identity is an important factor that affects students’ psychology and behavior. Individuals with a school identity have a higher sense of responsibility and academic achievement. A survey by Lian et al. (2021) found that school identity significantly negatively predicts learning burnout, and individuals who identify with the school show higher learning enthusiasm and initiative and less learning burnout. School identity is also affected by the school’s psychological environment. For example, Acosta et al. (2018) found that school psychological environment is closely related to school identity, and a positive school psychological environment promotes school identity. Studies have further shown that school identity is an important intermediary variable for predicting problematic behaviors in a school’s psychological environment (Zhai et al., 2020). Therefore, this study proposes the second hypothesis: school identity plays an intermediary role between the school psychological environment and learning burnout.

Learning burnout is affected not only by external situational factors (i.e., family, school, peers, etc.) but also by one’s own internal psychological factors (i.e., personality, motivation, sense of efficacy, etc.), both of which affect the generation of learning burnout (Zhang and Gao, 2019). As an important personality variable, self-esteem acts as an important protective factor that affects the physical and mental development of individuals. The theory of social identity proposes that self-esteem is divided into individual and collective self-esteem. The former is an individual’s positive evaluation of their own overall value, whereas the latter is an individual’s evaluation of the value and importance of their group and membership (Crocker and Luhtanen, 1990). In the context of Chinese collectivism culture, compared with individual self-esteem, collective self-esteem has a stronger collective and social color (Haslam et al., 2010) and has gradually become a focus area for researchers. Individuals are motivated to maintain and improve their level of collective self-esteem, and this motivation will bring more positive results (Peng et al., 2012). Empirical studies have also shown that collective self-esteem can improve an individual’s subjective well-being and sense of responsibility, while reducing depression and learning burnout (Brown and Hewstone, 2005). Collective self-esteem is closely related to a school’s psychological environment. Studies have found that a positive school mental environment significantly positively predicts collective self-esteem (Pfledderer et al., 2019). Good teacher-student relationships, sound infrastructure, and fair systems can encourage students to form a higher sense of belonging at school and make them feel that they are part of the school (Tong et al., 2018). In addition, the self-system process model also emphasizes that collective self-esteem, is an important part of the self-system, closely related to the individual’s environment; moreover, it is an important near-end factor affecting an individual’s psychological and behavioral development. This may be because of external environmental variables and development results, which plays an intermediary role (Connell and Wellborn, 1991). Therefore, this research proposes the third hypothesis: collective self-esteem plays a mediating role between school psychological environment and learning burnout.

According to the theory of self-categorization, when an individual forms an identity with a group, they will maintain or enhance the prestige and status of the group they belong to by comparing themselves with other outside groups, thereby obtaining a good collective self-concept and enhancing a positive self-image (Haslam et al., 2010). Empirical studies have shown that school identity and collective self-esteem are significantly positively correlated, and that and that an individual who identifies with the school will increase their collective sense of honor and pride and better adapt to the development of the school (Smith et al., 2020). Studies have also found that collective self-esteem is an important intermediary variable that affects college students’ sense of responsibility and psychological adaptation through school identity (Huang et al., 2016). Therefore, this study proposes the fourth hypothesis: school identity and collective self-esteem play a chain mediating role between school psychological environment and learning burnout.

**FIGURE 1:**
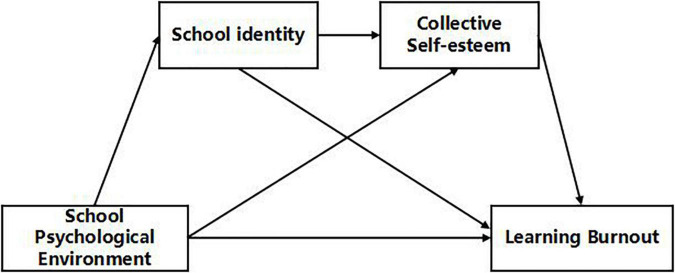
Hypothetical model: chain mediation model for the effect of school psychological environment on learning burnout via school identity and collective self-esteem.

In summary, the study considers medical students as participants, and explores the impact of school psychological environment on learning burnout; further, it examines the relationship between school identity and collective self-esteem based on the integration stage-environment matching theory, social identity theory, and self-categorization theory. The hypothetical model is shown in Figure 1.

## Materials and Methods

### Participants

Using convenience sampling method, we selected undergraduates from seven medical colleges and universities in the Anhui area. Further, 2,250 questionnaires were distributed and 2,031 valid questionnaires were returned, with an effective recovery rate of 90.27%. Among them, there were 569 first-year students (28.02%), 555 second-year students (27.33%), 437 third-year students (21.52%), 470 fourth-year students (23.14%) Overall 942 participants were men (46.38%) and 1,089 were women (53.62%); the participants’ age ranged between 17 and 23 years old, with an average age of 19.50 ± 1.41 years old.

### Measures

#### School Psychological Environment Questionnaire

The “University Student School Psychological Environment Questionnaire” (Xu and Zhong, 2019), which includes 30 items, was used to evaluate the state of school psychological environment as perceived by the participants. The questionnaire consists of six dimensions, including teacher-student relationships, classmate relationships, collective activities, professional development, resources, system, and order. In this study, we used a five-point scale for scoring, where 1–represents “never” and 5 which represents “always.” The higher the score, the more positive the psychological environment of the school. In this study, the Cronbach’s α coefficient of the questionnaire was 0.92.

#### School Identity Questionnaire

The “University Students’ School Identity Questionnaire” (Yang et al., 2020) was used to assess the individual’s level of school identity, which includes 20 items, such as group cognition, emotional connection, positive evaluation, and autonomous behaviors. A seven-point scoring method was adopted, ranging from 1–representing “completely disagree” to 7, which represented “completely agree.” The higher the score, the higher the degree of school recognition. In this study, the Cronbach’s α coefficient of the questionnaire was 0.94.

#### Collective Self-Esteem Scale

The “Collective Self-Esteem Scale” compiled by Luhtanen and Crocker (1992) consisting of 16 items was used to assess an individual’s level of collective self-esteem. The CSES includes eight inverted questions, via a seven-point scoring system, with 1–indicating “very inconsistent” and 7 “very consistent.” A higher score indicates a higher level of collective self-esteem. In this study, Cronbach’s α coefficient for the scale was 0.87.

#### Learning Burnout Scale

The College Student Burnout Scale (Lian et al., 2005) was used to assess an individual’s level of learning burnout. The LBS includes 20 items consisting of three dimensions: low mood, low sense of accomplishment, and misbehavior. It contains eight inverse questions and uses a five-point score rating, with 1 meaning “very inconsistent” and 5 “very inconsistent.” The higher the score, the higher the degree of learning burnout. In this study, the Cronbach’s α coefficient of the scale was 0.88.

### Procedure and Statistical Analysis

Collective tests were conducted based on a classroom like environment. The instructor served as the test leader, who introduced the instructions. Participants were asked to complete a form anonymously, based on their own experience or situations. It took approximately 10 min to complete all the questions, and all questionnaires were collected on the spot. SPSS 25.0 and AMOS 24.0 were performed for descriptive statistics, correlation analysis, and chain mediation effect tests.

## Results

### Common Method Bias and Multicollinearity Test

The results of Harman’s single factor method showed that a total of 15 factors with eight values greater than 1 were extracted, and the unrotated explained variance of the first factor was 28.44%, which was lower than the critical standard of 40% (Podsakoff et al., 2003). At the same time, the variance expansion factor values of all predictors ranged from 1.92 to 2.68 (≥5 indicates the presence of collinearity). Tolerance ranged from 0.37 to 0.52 (≤0.1 indicates the presence of collinearity) (O’ Brien, 2007). Thus, there was no serious common method deviation or multicollinearity in this study’s data.

### Descriptive Statistics and Correlations

The mean, standard deviation, and correlation matrix of each variable are listed in Table 1. Learning burnout and school psychological environment (*r* = −0.51, *p* < 0.001), school identity (*r* = −0.43, *p* < 0.001), and collective self-esteem (*r* = −0.56, *p* < 0.001) were significantly negatively correlated. School psychological environment and school identity (*r* = 0.64, *p* < 0.001), and collective self-esteem (*r* = 0.66, *p* < 0.001) were significantly positively correlated; moreover, school identity and collective self-esteem (*r* = 0.76, *p* < 0.001) were significantly positively correlated. As gender and grade significantly related to the main research variables, they were used as control variables in the subsequent analysis.

**TABLE 1:** Descriptive statistics and correlation analysis of the study variables.

Variable	1	2	3	4	5	6	7	8	9
1. Sex	1.00								
2. Age	0.03	1.00							
3. Grade 1	−0.07**	−0.64***	1.00						
4. Grade 2	−0.004	−0.16***	−0.38***	1.00					
5. Grade 3	0.02	0.25***	−0.33***	−0.32**	1.00				
6. School psychological environment	−0.01	−0.16***	0.23***	−0.06**	−0.04	1.00			
7. School identity	0.01	−0.13***	0.21***	−0.09***	−0.04	0.64***	1.00		
8. Collective self-esteem	0.03	−0.19***	0.28**	−0.10***	−0.09***	0.66***	0.76***	1.00	
9. Learning burnout	0.01	0.04	−0.14***	0.10***	0.03	−0.51***	−0.43***	−0.56***	1.00
M	0.54	19.50	0.28	0.27	0.22	3.44	5.06	4.87	2.68
SD	0.50	1.41	0.45	0.45	0.41	0.49	0.90	0.75	0.52

Gender and grade are categorical variables, so “gender” and “grade” were recoded into dummy variables before analysis (Guo et al., 2020). Among them, male = 0, female = 1; based on senior, Grade 1 = freshman vs. senior, Grade 2 = sophomore vs. senior, Grade 3 = junior vs. senior, ***p < 0.001 and **p < 0.01.

### Testing of the Mediation Effects From School Psychological Environment to Learning Burnout via School Identity and Collective Self-Esteem

**FIGURE 2:**
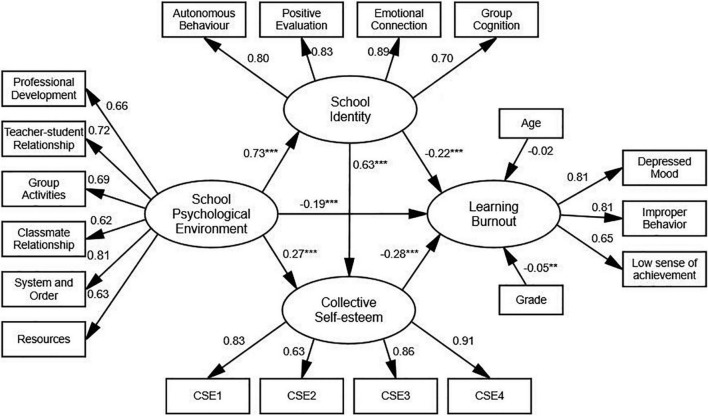
Structural equation model of effects of school psychological environment, school identity, and collective self-esteem on learning burnout. Controlled for age, grade as covariates. ***P < 0.001 and **P < 0.01.

Considering that the collective self-esteem scale has a single-dimensional structure and has many items, the balance method was used to package the data (Ma et al., 2017). The items are packaged into four observation variables (from collective self-esteem 1 to collective self-esteem 4). Using school psychological environment as an independent variable, school identity and collective self-esteem as intermediary variables, and learning burnout as a dependent variable, a structural equation model was established to test the mediating effect relationship between school identity and collective self-esteem in school psychological environment and learning burnout under the condition of controlling for grade and age. The results showed that the overall model fit well (χ^2^/df = 2.62, CFI = 0.94, TLI = 0.93, RMSEA = 0.07), and each path coefficient reached a significant level. As shown in Figure 2, school psychological environment significantly positively predicted school identity and collective self-esteem (β = 0.73, *p* < 0.001; β = 0.27, *p* < 0.001), significantly negatively predicted learning burnout (β = −0.19, *p* < 0.001), and significantly positively predicted collective self-esteem (β = 0.63, *p* < 0.001) and significantly negatively predicted learning burnout (β = −0.22, *p* < 0.001). Collective self-esteem also significantly negatively predicted learning burnout (β = −0.28, *p* < 0.001).

The bias-corrected non-parametric percentile bootstrap method was used to test the mediation effect, and the 95% confidence interval (CI) was calculated by repeatedly drawing 5,000 samples. If the CI does not contain 0, the indirect effect is significant (Wu and Wen, 2011). As shown in Table 2, the CI for the mediating effect of school identity and collective self-esteem between school psychological environment and learning burnout did not contain 0, indicating that the total indirect effect is significant. Specially, three indirect effect pathways influenced the relation of school psychological environment and learning burnout. Firstly, the mediating effect of “school psychological environment → school identity → learning burnout” was significant, indicating that school identity mediated the effects of school psychological environment on learning burnout. Secondly, the mediating effect of “school psychological environment → collective self-esteem → learning burnout” was significant, indicating that collective self-esteem mediated the effects of school psychological environment on learning burnout. Thirdly, “school psychological environment →school identity →collective self-esteem→ learning burnout” was significant, indicating that the relationship between school psychological environment and learning burnout mediated by the chain path of school identity and collective self-esteem.

**TABLE 2:** Standardized total, direct, indirect effects, and specific indirect effects of structural model.

Path	Effect	Effect ratio	95% CI
School psychological environment → School identity → Learning burnout	−0.16	28.57%	(−0.17, −0.15)
School psychological environment → Collective self-esteem → Learning burnout	−0.08	14.29%	(−0.11, −0.05)
School psychological environment → School identity → Collective self-esteem → Learning burnout	−0.13	23.21%	(−0.17, −0.09)
Total indirect effect	−0.37	66.07%	(−0.43, −0.31)
Direct effect	−0.19	33.93%	(−0.28, −0.10)
Total effect	−0.56		(−0.60, −0.50)

## Discussion

The results of this study showed that school psychological environment significantly negatively predicted learning burnout among medical students. This is consistent with the results of previous studies (Erceghurn and Mirosevich, 2008) and validates the first research hypothesis. This may be because a positive school psychological environment provides the necessary conditions for students’ learning. Individuals perceiving a safe, fair, and harmonious school environment are willing to spend time and energy learning and maintaining a positive attitude and interest in learning; this reduces learning burnout (Bush, 1979). In addition, a good teacher-student relationship and peer relationships on campus can stimulate students’ learning motivation, increase willingness to learn, and the likelihood of higher academic achievement (Gottfredson, 1975). This result is also in line with the stage-environment matching theory and the ecosystem theory. The growth and development of an individual cannot be separated from the environment in close contact with the individuals.

Individual-level variables should be nested within group-level variables. However, studies have found no significant difference between controlling group-level variables and multi-level analysis (Huang et al., 2016). In order to exclude the error of individual-level and group-level variables on the results, age and grade were treated as a control variable in our analysis process. Our study found that school psychological environment can reduce the learning burnout of medical students by improving their school identity. This result supports the situation-process-outcome model and validates the second hypothesis. Bush believes that school psychological environment consists of three types: interpersonal relationships (teacher-student relationships, peer relationships, etc.), personal development (professional development, collective activities, etc.), and system maintenance and change (system and order, resources, etc.) (Bush, 1979). The composition, which is in the midst of dynamic changes, has plasticity and controllability (Wang and Degol, 2016). Positive relationships in the school help to increase the school’s sense of identity and make students feel that they belong to the school and are connected to the school as a group (e.g., “I am proud to be a member of the school”; “I think I am a member of the school. Part of the school”) (Tong et al., 2018). According to social identity theory and self-categorization theory, when people identify with a certain group, they tend to internalize the group’s norms, values, and beliefs, which affects their psychology and behavior (Peng et al., 2012). Social control theory also proposes that a positive school atmosphere can strengthen the emotional connection between students and the school and promote ideal norms and behaviors, thereby reducing negative behavior (Prino et al., 2016). Therefore, when medical students perceive a safe and supportive school environment, it enhances the positive emotional connection with the school, increases the sense of belonging and pride, strengthens school identity, increases adherence to the school’s relevant regulations and requirements, stimulates more learning motivation and confidence, and facilitates active responses to the pressure of learning, thereby reducing learning burnout.

The study also showed that school psychological environment can reduce learning burnout of medical students by improving collective self-esteem, which validates the third hypothesis. This result also confirms the view of the self-system process model that external environmental variables will indirectly affect the results of individual development through the individual’s internal self-system (Connell and Wellborn, 1991). Teacher-student relationships and peer relationships are important dimensions of a school’s psychological environment. The harmonious interpersonal relationship in the school helps to create a warm and supportive atmosphere, allowing students to feel more supported to become more aware of their own group. The importance and value of the group will also hold yield more positive comments on other group members, showing a higher level of collective self-esteem (Hong et al., 2011). Additionally, the theory of terror management, suggests that self-esteem is an important factor in buffering anxiety (Burke et al., 2010). A meta-analysis of a longitudinal study also provides support for this theory, showing that both individual and collective self-esteem can significantly negatively predict anxiety (Sowislo and Orth, 2013). Anxiety is an important emotional manifestation of learning burnout. Individuals with high collective self-esteem tend to seek social support when facing difficulties, relieving anxiety and reducing learning pressure (Barker, 2009). Therefore, a positive school psychological environment can provide more physical and psychological support, improve medical students’ positive evaluation of their own group, while helping students form an optimistic attitude. Even if they are faced with learning pressure, it can be effectively alleviated through perceived support. This can then ensure they maintain sufficient learning input and motivation to reduce learning burnout.

This study also found that school identity and collective self-esteem play a chain-like mediating role between the school’s psychological environment and medical students’ learning burnout, which validates the fourth hypothesis. Previous studies have shown that collective self-esteem is an important explanatory variable for the influence of social identity on the psychological and behavioral patterns of its members. The formation of social identity can enhance positive self-image, self-esteem, and self-worth. School identity belongs to the social identity category. It refers to students’ emotional acceptance and affirmation of the school and its members. Individuals with higher school identity are more willing to actively participate in collective activities, experience mutual trust, cooperation, and respect within the group, and actively try to be identifiable in the group. This generates strong feelings of motivation and confidence, buffers bad emotions (Smith et al., 2020). Therefore, a positive school psychological environment can inspire a sense of belonging and identification with the school for medical students. This in turn facilitates adaptation to the school’s rules and regulations and developmental requirements, enhances awareness of their values and responsibilities in the school, and strengthens learning motivation to overcome the pressure of learning, ultimately reducing learning burnout.

Besides, the cross-sectional study has the advantages of saving time and money, being easy to implement, and having a low rate of attrition (Wang and Cheng, 2020), and its practical effect has been confirmed in a large number of empirical studies (Wang et al., 2018; Khoury-Kassabri et al., 2020; Jing et al., 2021). Compared with cross-sectional research, longitudinal research can better reveal the causal relationship between variables, but considering long-term fixed sample tracking may face problems such as missing subjects and test-retest effects, which affect the validity of the results (Ma, 2010). Therefore, this study considered the actual situation such as time and subject conditions, and did not use longitudinal research to collect data.

In conclusion, results highlight the effects of preventing and reducing learning burnout among medical students. Attention should be given to the role of the school’s psychological environment and the creation of a supportive school atmosphere. Schools and teachers can start by improving teacher-student relations and improving infrastructure and learning resources to provide students with a comfortable and healthy external environment for reducing learning burnout. These actions will also potentially improve students’ sense of identity and belonging to the school. At the school level, publicity and reports on the advantages and characteristics of the school should be developed to enhance the school’s reputation and influence. Further, at the student level attention should be paid to school development and discovering its advantages, gratefully experience its humanistic care for students. In addition, a collective self-esteem should be cultivated. Teachers should be encouraged to actively organize and implement thematic group activities, construct a more cohesive sense of class and dormitory culture, and enhance the sense of belonging by honoring student achievements and the fostering of social integration the school and class.

### Limitations

This study chose counselors as the test may affect the results to a certain extent, future research should adopt a double-blind design to further reduce the measurement errors. Besides, Longitudinal studies may better reveal causal relationships between variables, future studies should consider the use of longitudinal study designs.

## Data Availability Statement

The raw data supporting the conclusions of this article will be made available by the authors, without undue reservation.

## Ethics Statement

The studies involving human participants were reviewed and approved by the Biomedical Ethics Committee of Anhui Medical University. Written informed consent to participate in this study was provided by the participants’ legal guardian/next of kin.

## Author Contributions

WYu and JB: conceptualization. WYa, SY, and MC: methodology and formal analysis. WYu: writing—original draft preparation. WYa, JB, YZ, and QM: writing—review and editing. All authors have read and agreed to submit the manuscript.

## Conflict of Interest

The authors declare that the research was conducted in the absence of any commercial or financial relationships that could be construed as a potential conflict of interest.

## Publisher’s Note

All claims expressed in this article are solely those of the authors and do not necessarily represent those of their affiliated organizations, or those of the publisher, the editors and the reviewers. Any product that may be evaluated in this article, or claim that may be made by its manufacturer, is not guaranteed or endorsed by the publisher.
